# Anti-microbial Antibodies, Host Immunity, and Autoimmune Disease

**DOI:** 10.3389/fmed.2018.00153

**Published:** 2018-05-23

**Authors:** Peilin Zhang, Lawrence M. Minardi, J. Todd Kuenstner, Steven M. Zekan, Rusty Kruzelock

**Affiliations:** ^1^PZM Diagnostics, LLC, Charleston, WV, United States; ^2^WV Regional Technology Park, South Charleston, WV, United States

**Keywords:** anti-microbial antibody, autoimmune disease, molecular mimicry, host immunity, autoantibody

## Abstract

Autoimmune diseases are a spectrum of clinical inflammatory syndromes with circulating autoantibodies. Autoimmune diseases affect millions of patients worldwide with enormous costs to patients and society. The diagnosis of autoimmune diseases relies on the presence of autoantibodies and the treatment strategy is to suppress the immune system using specific or non-specific immunosuppressive agents. The discovery of anti-microbial antibodies in the blood of patients with Crohn's disease and Sjogren's syndrome and cross-reactivity of anti-microbial antibodies to human tissue suggests a new molecular mechanism of pathogenesis, raising the possibility of designing a new therapeutic strategy for these patients. The presence of anti-microbial antibodies indicates the failure of the innate immunity system to clear the microbial agents from the blood and activation of adaptive immunity through B-lymphocytes/plasma cells. More importantly, the specific antibodies against the microbial proteins are directed toward the commensal microbes commonly present on the surface of the human host, and these commensal microbes are important in shaping the development of the immune system and in maintaining the interaction between the human host and the environment. Persistence of these anti-microbial antibodies in patients but not in normal healthy individuals suggests abnormal interaction between the human host and the commensal microbes in the body. Elimination of the organism/organisms that elicits the antibody response would be a new avenue of therapy to investigate in patients with autoimmune diseases.

## Introduction

Autoimmune diseases are a group of systemic inflammatory syndromes characterized by the presence of autoantibodies in the circulation (1) with exception of Crohn's disease (CD). The spectrum of autoimmune diseases is large involving many different organ systems, and the autoantibodies are diverse in their biochemical properties. Although many autoantibodies are identified in different diseases, the molecular mechanisms of these autoantibodies in pathogenesis and disease progression is largely unknown. It's reasonably clear that all autoimmune diseases occur in genetically susceptible individuals, and human susceptibility gene discovery is important in uncovering the pathogenesis (2). Indeed, many genetic susceptibility loci are found in autoimmune diseases. Many genetic loci in autoimmune diseases are related to the innate immunity network, but how the innate immunity network links to pathogenesis is yet to be defined. Recent advances in the field of the human microbiome have dramatically enhanced our understanding of the symbiotic relationship between the host and its microbiota. It is now clear that the human surface, external, and internal, are covered by a huge number of diverse bacteria, and these bacteria are important in the development of host immunity, pathogen invasion, immune tolerance, and metabolism (3, 4). The relationship of these microbes to disease processes are gradually emerging, and our discovery of anti-microbial antibodies cross-reacting to human tissues suggests a new mechanism of pathogenesis with the potential to design a new therapeutic strategy for a broad spectrum of autoimmune diseases (5). Some of the bacteria in the gut and on the skin which are not currently recognized as pathogens in the human host may be eliciting the antibody response and causing disease in the host. The finding of the anti-microbial antibodies and cross-reactivity to human tissue reminds us of the history of Streptococcal laryngitis followed by rheumatic valvular heart disease and glomerulonephritis (6–8). Both rheumatic valvular heart disease and glomerulonephritis are dramatically decreased in incidence in the US due to the adoption of new therapeutic and preventive measures for Streptococcal infection. Similarly, the presence of anti-microbial antibodies within the circulation of patients with autoimmune diseases offers a new opportunity for therapeutic design for a large spectrum of autoimmune diseases.

## Innate immunity and adaptive immunity:

Human immunity is classified as innate and adaptive. Innate immunity consists of cell-mediated and humoral mediated immunity and also includes physical barriers such as skin and mucosa that prevent pathogens from entering the human body. The innate immunity system in the blood includes phagocytes (neutrophils, macrophages, eosinophils, and basophils), mast cells, and the immunoglobulin/complement system. The innate immune response is immediate, maximal, and non-specific with no immunologic memory and is a basic defense system in all forms of life. Adaptive immunity, on the other hand, is pathogen and antigen specific with lag time from the exposure to the maximal response. It is related to specific antibody production and it leads to immunological memory. Bacterial immunity is complicated and the pathogenic bacteria are mostly removed from the blood circulation by the innate immunity system including neutrophils, macrophages, and the complement system. Under normal circumstances, bacterial clearance from the blood does not require antibody production. Unlike clearance of viruses in which specific adaptive immunity such as specific antibody development is required, bacterial clearance is non-specific, and typically by two separate mechanisms. First, the phagocytes including neutrophils, macrophages, eosinophils, or mast cells can engulf the bacteria through endocytosis, lysosomal fusion, and autophagy to destroy the bacteria and clear the bacteria from blood. Second, bacteria express specific surface proteins, such as protein A in *S. aureus*, protein G in *Streptococcus*, protein L in *Clostridium* and protein D in *E.coli*, and these bacterial proteins can bind non-specifically to immunoglobulin with high affinity so the immunoglobulin/bacteria complex can be cleared by the complement system and macrophages (opsonization) (9–11). No adaptive immunity is required in the clearance of common bacterial infection. It is now known that there are a large number of commensal bacteria commonly present on the surface of the human host, and these commensal bacteria play important roles in human immune development, immune tolerance, and defense of the human host from invasion of pathogens (3). Commensal bacteria are non-pathogenic to the host due to the physical barrier system with close interaction between the epithelial cells, immune cells (lymphocytes), and the microbes (12, 13). The fact that patients with various diseases develop specific antibody against the commensal bacteria suggest two important points: there is a defect(s) in innate immunity including the cellular components and immunoglobulin/complements components, to clear the bacteria from the blood effectively, or the bacteria lack expression of the specific protein due to genetic mutations, such as protein A deficient *S. aureus* or both (14). Mycobacterium avium subspecies paratuberculosis (MAP) or a similar pathogen, cannot be cleared by the host and in the case of CD, the host has persistent mucosal lesions, and thus an ineffective barrier that cannot prevent commensal bacteria from entering the circulation. The constant exposure to these commensal bacteria in the circulation may lead to an antibody response to these commensal bacteria. Specific defects of innate immunity including the physical barrier and complements with specific bacterial infections have been previously described [see reviews (15, 16)]. These previous studies are more dramatic with the phenotype of complete lack of genes important for innate immunity (17), but less pronounced in the vast majority of cases of clinical disease in which the patient does not completely lack these genes/gene products. Bacterial genetic mutations are well-documented in antibiotic resistance but why these bacteria infect human blood (culture-positive sepsis) without being cleared by the innate immune system has not been widely-studied.

## Identification of antimicrobial antibodies:

We are interested in finding infectious agents in Crohn's disease (CD) patients through blood culture, molecular identification, and serology to assess the host response to infectious agents with an emphasis on MAP (18). In our experience, the culture method described in reference 18 had a low yield of MAP positive specimens, but the serology testing results indicated over 70% of our Crohn's patients are positive for MAP antibody in their blood using the whole MAP cell extract as capturing antigens. We have also discovered the DNA of many different bacteria in the blood of CD patients by PCR and sequencing (see below). The discrepancy between the results of molecular PCR and MAP antibody serology is so dramatic that prompted us to investigate the cause of the reaction of human plasma from CD patients to MAP extracts. Since we and others have found a diversity of bacterial DNA in the blood, we included other bacteria in our analysis (19, 20). We analyzed the bacterial extracts by Western blot using plasmas of patients with CD, similar to previous studies in the 1980s and 1990s (21, 22). We found specific reactivity of patients' plasmas to specific microbial proteins of commensal bacteria commonly present on the surface of our body and in the environment (23). The discovery of specific antibody in human plasma against the commensal bacteria such as *Staphylococcus aureus* and *Escherichia coli* in CD and SS patients but not in controls is counter-intuitive, since the human body is developed with a complex defense mechanism as described above. These antimicrobial antibodies are only present in the patients, but not in the normal healthy individuals. A brief description of the discovery of anti-microbial antibodies from a variety of patients with autoimmune diseases follows.

## Specific antibodies against microbial proteins

Our laboratory discovered 7 microbial proteins reactive to the plasma from the patients with CD and SS (Table 1) (23). We identified these microbial proteins from various bacteria through Western blot analysis, immunoprecipitation using the patient's plasma as primary antibodies, and LC mass spectrometry. We confirmed the specific interaction between human antibodies and the bacterial proteins by three separate methods. First, we used human plasma from the patients as primary antibodies to incubate with the bacterial extracts separated on the Western blot analysis to determine the specific bacterial proteins reactive to the patient's antibody (23). Second, we used the specific antibodies against the human homolog of bacterial proteins as primary antibodies to incubate with bacterial extracts separated on the Western blot analysis (not shown). Third, we used the specific anti-bacterial antibodies to incubate with human tissue on the microscopic slides for specific reaction to specific human tissues using immunohistochemical staining. There are four bacterial proteins from the *Staphylococcus* family, although *Staphylococcus* plays no significant roles in the pathogenesis of CD to date. We and others have identified a variety of bacterial DNA from the *Staphylococcus* family in the blood of CD patients, and the role of *Staphylococcus* in CD is yet to be defined (19, 24). *S. aureus* and *Streptococcus* may be important for SS. *E.coli* is present in the gut in abundance and it may play a role in the pathogenesis of CD and ulcerative colitis (25–27). Hsp65 (GroEL) is found commonly in the Mycobacterium family, and similar proteins are found in many other bacterial species including *Borrelia* species in Lyme disease (27, 28). The p58 in Western blot analysis for the diagnosis of Lyme disease is an identical protein to hsp65 in mycobacteria (29).

**Table 1:** Microbial proteins reactive to plasmas from Crohn's and Sjogren's patients.

**Name**	**MW(Kd)**	**Microbial species**
DNA-directed RNA polymerase subunit B (RPOB)	135	*S. aureus*
Elongation factor G (EF-G)	75	*S. aureus/ pseudintemedius*
ATP synthase subunit alpha (ATP5a)	55	*S. aureus/ pseudintemedius*
Uncharacterized lipoprotein SACOL0083 (unknown)	27	*S. pseudintemedius*
Elongation factor Tu (EF-Tu)	42	*Escherichia coli*
Outer membrane porin protein C (ompC)	39	*Escherichia Coli*
Heat shock protein 65 (hsp65)	60	*M. paratuberculosis*

Human homologs of the bacterial proteins exist for four of these seven proteins, suggesting conservation of these proteins across the species. Interestingly, three of the human homologs, EF-G, ATP5a, and EF-Tu, are mitochondrial proteins. Polyclonal antibody against the bacterial EF-Tu can react to human tissue, but the reactivity signal is nuclear rather than cytoplasmic (mitochondrial). It is unclear if the mitochondrial function in the patients with CD or autoimmune diseases is impaired, but chronic fatigue is one of the most pronounced features in all autoimmune diseases, and the energy metabolism of the mitochondria and the detoxification function of mitochondria should be further examined. Mitochondrial dysfunction appears important in many neurodegenerative disorders. *ATP5b autoantibody was found in both the Guillain-Barre syndrome and Alzheimer's disease* (30, 31). It would be interesting to study these relationships between the bacterial proteins and the human mitochondrial functions in various neurodegenerative disorders.

The observation that specific anti-microbial antibodies react to human tissue is important, and helps explain the pathogenesis of autoimmune disease, considering that the function of most autoantibodies is essentially unknown. For example, the presence of rheumatoid factor (RF) in rheumatoid arthritis (RA) is well recognized, and RF factor serves as a diagnostic biomarker for RA (32). RF is an autoantibody against the Fc region of immunoglobulin G. An explanation for the development of RF and the relationship of RF to the damage of joint cartilage/bone is unclear. It is entirely possible that RF is produced through molecular mimicry to a specific protein epitope of virus or bacterium.

Hsp65 of *Mycobacterium tuberculosis* is highly homologous to human hsp60 (33), and it is not clear whether hsp60 autoantibody in many clinical diseases such as cancer, and autoimmune diseases are the result of cross reactivity to hsp65 of mycobacteria, since mycobacteria, pathogenic or non-pathogenic, are abundant in the environment (27, 33). Through all mycobacterial species, hsp65 is well conserved, and our data indicate many patients with CD and SS are positive for Hsp65 antibody by ELISA assay (23). The presence of anti-hsp65 antibody in patients with CD and SS but not in the normal healthy control population suggests a previous exposure to mycobacterium in the blood. *Recent work from Sharp et al implicates MAP as a possible cause of RA* (34). In order for bacteria/mycobacteria to enter the blood circulation, the physical barriers such as skin or mucosa have probably been damaged and a transient/persistent bacteremia/mycobacteremia has occurred. Again, the blood clearing mechanism, *either the barrier dendritic cells, or T-cells, or neutrophils, etc., has* to fail so that the adaptive immunity subsequently can be activated to produce specific anti-microbial antibodies. Persistent elevation of antibody levels in blood suggests a disease state since the antibody level will fade over time, if no new stimulus is constantly present.

Our data indicate that patients with CD and SS have antibodies to MAP and antibodies against commensal bacteria commonly present on the human body. These commensal bacteria are abundant. We also showed these antibodies can cross react to human tissue in similar fashion to Streptococcal antibody and myosin of cardiac muscle (35, 36). If these data can be independently verified, a new molecular mechanism of pathogenesis of autoimmune diseases emerges, and an infectious/microbial trigger in autoimmune diseases becomes plausible. The close symbiotic relationship between the commensal bacteria and the human host appears disrupted in the disease state, and these bacteria start to penetrate the barrier to enter the blood circulation. It is apparently a scenario of “the enemy within,” and the host defense system (specific antibodies) causes collateral damage in fighting the invading pathogens in these autoimmune diseases.

## Antibiotics and autoimmune diseases

There are two different arguments regarding the use of antibiotics in autoimmune diseases. There is a growing body of literature arguing the use of antibiotics or certain classes of antibiotics in autoimmune diseases, and the aminoglycosides can induce production of auto-antigens leading to production of autoantibodies, a hallmark of autoimmune disease (37). Antibiotic use has been linked to increased risk of inflammatory bowel disease in children exposed to broad spectrum antibiotics (38, 39), and it has been shown to modulate human HLA genes and type I diabetes (37). However, there is an equally large body of literature using various antibiotics for therapy of autoimmune diseases (40–42). Certain antibiotics such as hydroxychloroquine (Plaquenil) possess unique characteristics to inhibit inflammation and it is widely used to treat SLE. Many small trials and anecdotal case report support the use of antibiotics in CD and in various autoimmune diseases (43). Antibiotics have been shown to reduce the risk of rheumatoid arthritis in mice (44). Finally, Redhill Biopharma is currently conducting a large clinical trial of triple antibiotics in CD, but previous similar trials provided inconclusive results and clinical guidelines in this area are controversial (42, 43).

Based on our findings of circulating anti-microbial antibodies in patients with various autoimmune diseases, and that these antibodies are produced by the presence of constant stimuli from the commensal microbes, it is conceivable that a short term course of antibiotics to remove the microbial stimuli may benefit some patients with autoimmune diseases. But long term use of antibiotics must be analyzed for risk/reward, since all antibiotics have significant adverse effects. Our data demonstrate that specific antibodies against a specific species of bacteria are present in certain patients, and broad spectrum antibiotics may be considered instead of narrow–spectrum antibiotics without testing the antibodies in the patients. A simple serology test can identify specific antibodies against specific bacteria and antibiotics can be tailored for the patients.

Our data in CD and SS patients and Sharp's data in RA patients showing evidence of MAP infection combined with the open label trials and anecdotal reports in CD showing resolution of disease with clearance of MAP bacteremia suggests new therapeutic avenues for the treatment of SS and RA. If MAP bacteremia is demonstrable in SS and RA patients, will their disease resolve if the MAP bacteremia is cleared? MAP prevalence studies and clinical trials with anti-MAP therapy in this population may answer these questions.

## Conclusion

We have found anti-microbial antibodies in autoimmune diseases and shown that these antibodies cross react to human tissue. Those clinically important autoantibodies may represent anti-microbial antibodies against commensal bacteria that enter the circulation through damaged physical barriers. The microbiota is different in various organs and the specific microbiota may explain the variety of autoantibodies and various disease processes. This new paradigm represents a new frontier with opportunities to improve the diagnosis and treatment of autoimmune diseases.

## Author contributions

PZ wrote the manuscript. JK edited the manuscript. All authors reviewed the manuscript.

### Conflict of interest statement

PZ, LM, JK, and SZ are stake owners PZM Diagnostics, LLC and are affiliated financially with the company. RK declares no competing interest.
